# Distinct Roles for ROCK1 and ROCK2 in the Regulation of Keratinocyte Differentiation

**DOI:** 10.1371/journal.pone.0008190

**Published:** 2009-12-04

**Authors:** Frances E. Lock, Neil A. Hotchin

**Affiliations:** School of Biosciences, University of Birmingham, Birmingham, United Kingdom; Louisiana State University, United States of America

## Abstract

**Background:**

The human epidermis is comprised of several layers of specialized epithelial cells called keratinocytes. Normal homoeostasis of the epidermis requires that the balance between keratinocyte proliferation and terminal differentiation be tightly regulated. The mammalian serine/threonine kinases (ROCK1 and ROCK2) are well-characterised downstream effectors of the small GTPase RhoA. We have previously demonstrated that the RhoA/ROCK signalling pathway plays an important role in regulation of human keratinocyte proliferation and terminal differentiation. In this paper we addressed the question of which ROCK isoform was involved in regulation of keratinocyte differentiation.

**Methodology and Principal Findings:**

We used RNAi to specifically knockdown ROCK1 or ROCK2 expression in cultured human keratinocytes. ROCK1 depletion results in decreased keratinocyte adhesion to fibronectin and an increase in terminal differentiation. Conversely, ROCK2 depletion results in increased keratinocyte adhesion to fibronectin and inhibits terminal differentiation.

**Conclusion:**

These data suggest that ROCK1 and ROCK2 play distinct roles in regulating keratinocyte adhesion and terminal differentiation.

## Introduction

The human epidermis is comprised of several layers of specialized epithelial cells called keratinocytes. As keratinocytes are lost from the outermost epidermal layers, they are replaced through a process of terminal differentiation in which keratinocytes in the basal layer exit the cell cycle, down-regulate adhesion to the extracellular matrix (ECM) proteins of the basal lamina and migrate upwards through the supra-basal, differentiated layers, until they eventually reach the outermost cornified layer [1]. The basal lamina is made up of various ECM proteins, including fibronectin, collagens and laminins. Keratinocytes in the basal layer of the epidermis adhere to these ECM proteins via integrin adhesion receptors and there is considerable evidence that adhesion to ECM plays a key role in regulating epidermal function [1]. Disruption of integrin-ECM interactions results in initiation of keratinocyte terminal differentiation in vitro [1]–[3]. Hence, normal epidermal function requires that the balance between keratinocyte proliferation, adhesion to ECM proteins and terminal differentiation be tightly regulated. Previous data from our laboratory and others suggest that signalling though Rho family GTPases is required for keratinocyte terminal differentiation [4]–[6]. RhoA is a member of the Rho family of small GTPases and acts as a molecular switch to regulate a plethora of cellular processes including organisation of the actin cytoskeleton, cell adhesion and motility and gene expression [7]. The best-characterised downstream effectors of RhoA are the serine/threonine kinases ROCK1 and ROCK2 (also known as ROKβ and ROKα, respectively) [8], [9]. Both ROCK isoforms are comprised of an N-terminal region, a kinase domain, a coiled-coil domain containing a Rho binding site, a PH domain and a C-terminal domain [10]. Both isoforms share a high amino acid sequence identity, with 92% identity across their kinase domains. However, the two kinases only share 65–70% sequence identity across their PH domains, which may account for the observed differences in cellular localisation of the two isoforms [8], [9], [11]. Most studies to date have either used over-expression of ROCK or pharmacological inhibition of ROCK [4], [12], [13]. Neither of these methods allows discrimination of isoform-specific functions. Recently, functional differences between the two ROCK isoforms have become more apparent. In vivo data show that, despite their structural similarities, ROCK1 or ROCK2 expression cannot compensate for loss of the other isoform during murine embryonic development [14]–[16]. In vitro studies utilising ROCK isoform specific RNAi knockdown in fibroblasts also suggest that ROCK1 and ROCK2 may have distinct, and sometimes opposing, roles in the cell [11], [17]. In this study we used RNAi to specifically knockdown ROCK1 or ROCK2 expression in cultured keratinocytes and analysed adhesion to various ECM proteins and the differentiation status of the cells. Our data suggest that both ROCK isoforms play distinct and important roles in regulating keratinocyte differentiation status and keratinocyte adhesion to the ECM protein fibronectin.

## Results

**Figure 1:**
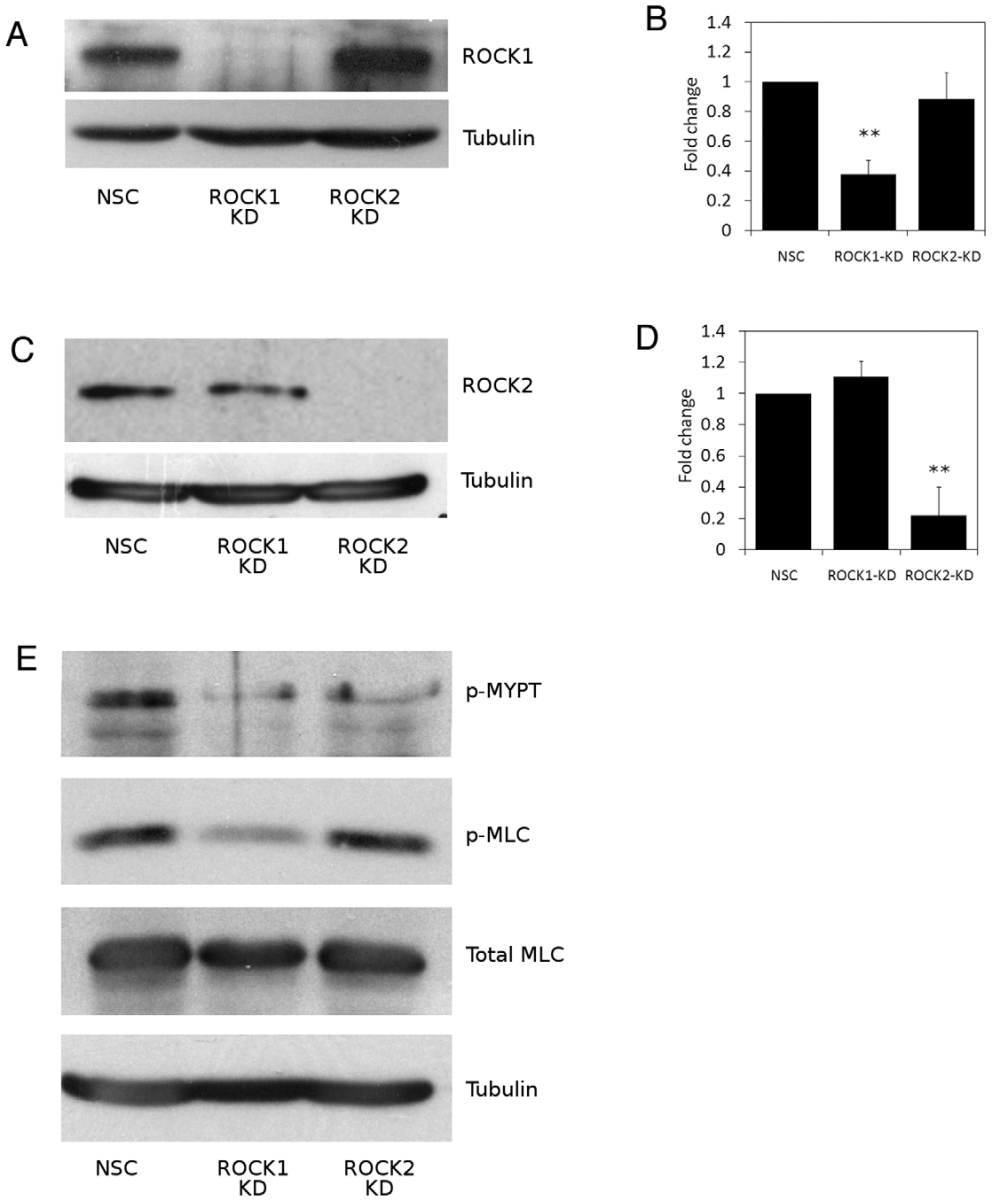
ROCK isoform-specific knockdown affects phosphorylation of downstream targets. HaCaT-NSC, HaCaT-ROCK1-KD or HaCaT-ROCK2-KD cell lysates were immunoblotted to assess ROCK1 expression (A) or ROCK2 expression (C). B and D show densitometric analysis of knockdown of ROCK1 (B) or ROCK2 (D) relative to non-silencing (NSC) control cells and are the mean of 3 separate experiments (** p<0.01). Phosphorylation of myosin phosphatase-1 residue Thr696 (p-MYPT) and myosin light chain residue Ser19 (p-MLC) were also analysed and data shown are representative of 3 separate experiments (E).

HaCaT keratinocytes were stably transfected with GFP-IRES-shRNAmir constructs specifically targeting ROCK1 or ROCK2 or a non-silencing control nonsense mRNA sequence (NSC) to generate HaCaT-ROCK1-KD, HaCaT-ROCK2-KD and HaCaT-NSC cells respectively. A stable decrease in ROCK1 expression was observed in HaCaT-ROCK1-KD cells, compared to HaCaT-NSC and HaCaT-ROCK2-KD cells (Figure 1A, B). Similarly, a significant decrease in ROCK2 expression was observed in HaCaT-ROCK2-KD cells, when compared to HaCaT-NSC and HaCaT-ROCK1-KD cells (Figure 1C, D). Depletion of ROCK1 or ROCK2 had no effect on expression of the other, non-targeted, ROCK isoform (Figure 1A, C). To further characterise these cell lines following ROCK isoform knockdown, HaCaT-NSC, HaCaT-ROCK1-KD and HaCaT-ROCK2-KD cell lysates were immunoblotted to assess changes in phosphorylation of two known ROCK targets - myosin phosphatase (MYPT) and myosin light chain (MLC). Both ROCK1 and ROCK2 are able to directly phosphorylate MYPT1 on threonine residue 696 whereas serine 19 of MLC is phosphorylated by ROCK1 but not ROCK2 [18]–[20]. Decreased MYPT phosphorylation was observed in both HaCaT-ROCK1-KD and HaCaT-ROCK2-KD cells when compared to HaCaT-NSC cells (Figure 1E). Consistent with MLCpSer19 being a ROCK1 substrate but not a ROCK2 substrate, a decrease in phosphorylated MLC was observed in HaCaT-ROCK1-KD cells, but not in HaCaT-ROCK2-KD or HaCaT-NSC cells (Figure 1E). These results confirm that stable knockdown of ROCK1 and ROCK2 expression in these HaCaT keratinocyte cell lines is isoform specific and has functional consequences in terms of phosphorylation of known downstream effectors.

**Figure 2:**
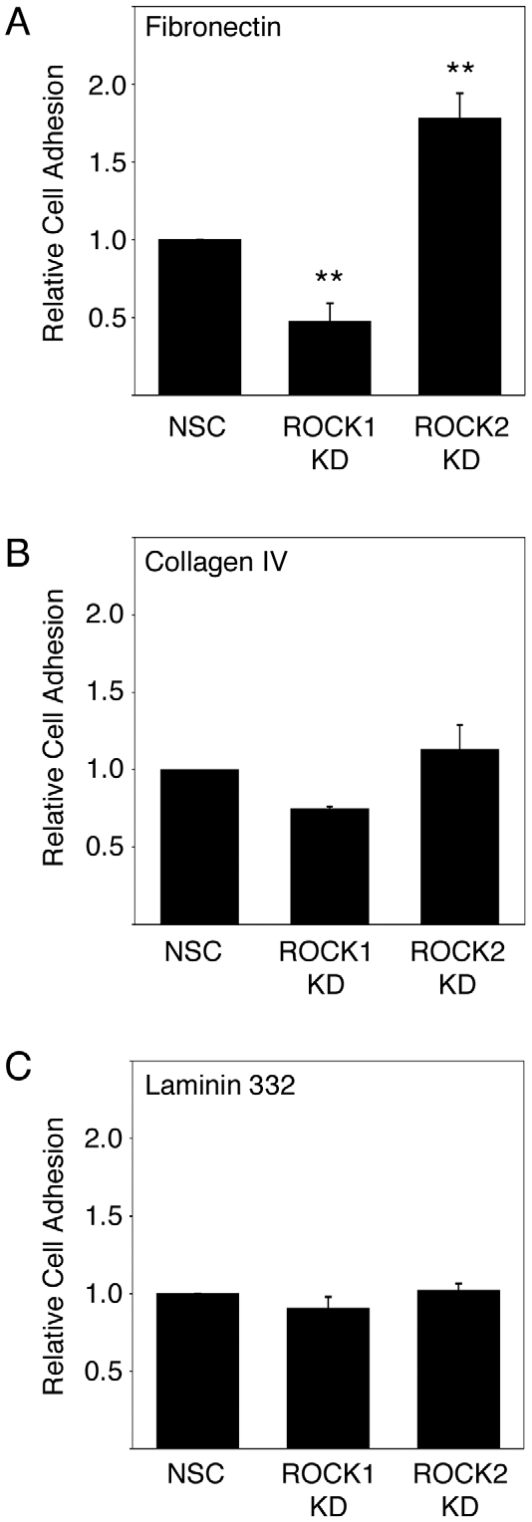
ROCK isoform-specific knockdown regulates cell adhesion to fibronectin. Adhesion of HaCaT-NSC, HaCaT-ROCK1-KD or HaCaT-ROCK2-KD to the extracellular matrix ligands fibronectin (A) collagen IV (B) and laminin 332 (C) was analysed. The mean and standard error of 3 separate experiments are shown in each case. Statistical analysis was carried out using unpaired two-way Student's T-test (** p<0.01).

Previous work has established clear functional links between adhesion to fibronectin, integrin signalling and keratinocyte terminal differentiation. [1], [2], [21]. To assess the role of ROCK1 and ROCK2 in keratinocyte adhesion we analysed adhesion to various extracellular matrix proteins. Following ROCK1 depletion, a significant decrease in keratinocyte adhesion to full-length fibronectin was observed when compared to HaCaT-NSC cells (Figure 2A). In contrast, ROCK2 depletion resulted in a significant increase in cell adhesion to fibronectin (Figure 2A). Similar results were observed using recombinantly expressed FIII9-10 integrin-binding domain of fibronectin as a ligand (Figure S1). Adhesion to the ECM proteins collagen IV and laminin-332 (previously called laminin V) was also assessed. Both are known keratinocyte ligands, but no significant differences in HaCaT adhesion were observed following ROCK1 or ROCK2 knockdown, when compared to HaCaT-NSC cells (Figure 2B, C). Over the duration of the assay no obvious differences in cell morphology were observed between any of the cell lines (data not shown).

**Figure 3:**
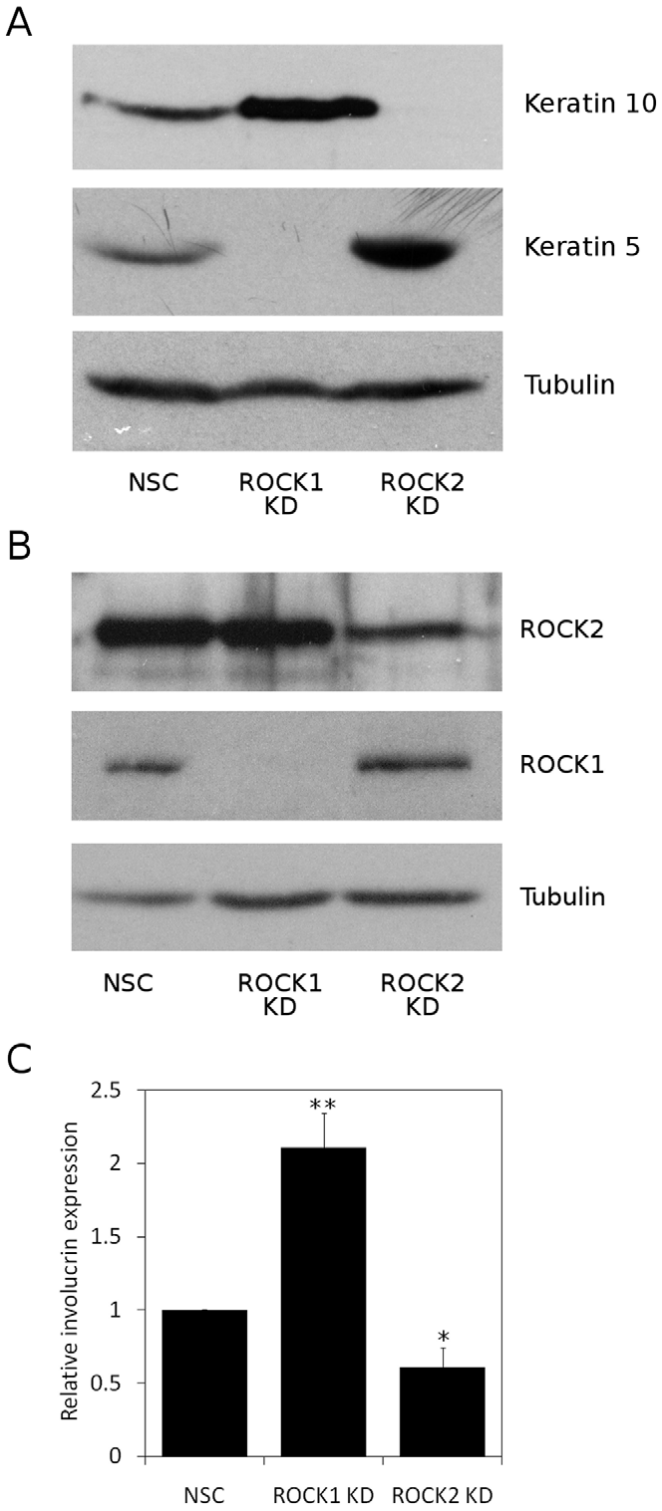
ROCK isoform-specific knockdown regulates keratinocyte differentiation. A, HaCaT-NSC, HaCaT-ROCK1-KD or HaCaT-ROCK2-KD cells were cultured for 2 days post-confluence and lysed. Expression of keratin 5, keratin 10 and tubulin were assessed by immunoblotting (A). SCC12f keratinocytes were transiently transfected with siRNA oligos to specifically knockdown ROCK1 or ROCK2 As a control SCC12f cells were transfected with a non-silencing control oligo (NSC). Cells were lysed and immunoblotted to assess ROCK isoform knockdown (B) or fixed and immunostained to assess involucrin expression (C). The means and standard errors from 3 separate experiments are shown. Statistical analysis was carried out using unpaired two-way Student's T-test, ** p<0.01, * p<0.05.

Having observed clear differences in cell adhesion to fibronectin between ROCK1 and ROCK2 depleted keratinocytes, we assessed the differentiation status of both cell lines. HaCaT-NSC, HaCaT-ROCK1-KD and HaCaT-ROCK2-KD cells were cultured for 2 days post-confluence to induce differentiation and cells lysed and immunoblotted to assess expression of basal and suprabasal keratins. Loss of the basal keratinocyte marker, keratin 5, is observed during keratinocyte differentiation and expression of keratin 5, was greatly reduced following ROCK1 depletion when compared to HaCaT-NSC cells (Figure 3A). Increased expression of keratin 10, a supra-basal marker of keratinocyte terminal differentiation, was also observed in HaCaT-ROCK1-KD cells (Figure 3A). Conversely, expression of keratin 5 was increased, and expression of keratin 10 was decreased in HaCaT-ROCK2-KD cells, when compared to HaCaT-NSC cells (Figure 3A). This would be consistent with an inhibition of keratinocyte terminal differentiation following ROCK2 depletion. To further characterise this differentiation phenotype we used different RNAi methodology and an alternative keratinocyte cell line to analyse spontaneous differentiation in sub-confluent cells. SCC12f cells are derived from a squamous cell carcinoma but grow and differentiate in a manner similar to that seen in normal primary keratinocytes and are a well-established model for keratinocyte function [22]. We transiently transfected SCC12f keratinocytes with siRNA oligos targeted against ROCK1 and ROCK2. The target sequences used were different to those targeted using the shRNAi vectors. As a control, SCC12f cells were also transfected with non-silencing control oligos (NSC). Following transient transfection with siRNA oligos, sub-confluent SCC12f cells were cultured, lysed and immunoblotted. As shown in Figure 3B, we observed isoform-specific knockdown of ROCK1 and ROCK2 in SCC12f cells although the knockdown of ROCK2 was not complete. To assess the rate of spontaneous differentiation in these cells, expression of the cornified envelope precursor involucrin, a commonly used marker for differentiation, was analysed. Transiently transfected, sub-confluent, SCC12f were fixed and the percentage of cells expressing involucrin was quantified by immunocytochemistry. siRNA-mediated knockdown of ROCK1 expression resulted in a 2-fold increase in the percentage of cells expressing involucrin, when compared to NSC controls (Figure 3C). In contrast, knockdown of ROCK2 expression resulted in a significant decrease in the percentage of involucrin expressing cells (Figure 3C and Figure S2). These data are consistent with the changes in keratin 5 and keratin 10 expression observed in HaCaT-ROCK1-KD and HaCaT-ROCK2-KD cells (Figure 3A). Taken together, these data suggest that loss of ROCK1 expression promotes keratinocyte terminal differentiation and that loss of ROCK2 expression has the opposite effect, inhibiting keratinocyte terminal differentiation.

## Discussion

Our data confirm that both ROCK isoforms are expressed in cultured human keratinocytes and that each isoform can be specifically depleted, with no effect on the expression of the other (Figure 1A–D). We observed that depletion of either ROCK1 or ROCK2 results in decreased phosphorylation of MYPT on Thr696 (Fig 1E). This is consistent with published data where both ROCK1 and ROCK2 have been reported to phosphorylate MYPT on Thr696, leading to its inactivation [18]–[20]. In contrast, we observed a specific loss in phosphorylation of MLC on Ser19 in ROCK1 depleted cells but not in ROCK2 depleted cells (Figure 1E). Again, this is consistent with recent studies describing MLC as a ROCK1-specific target [11], [14], [23]. This implies that continued expression of one isoform cannot compensate for the loss of the other, suggesting specific functional differences in human keratinocytes. Previous work from our laboratory has shown that the Rho/ROCK signalling pathway is important in regulating keratinocyte function [4]. Here we have shown that the two ROCK isoforms have distinct roles in the regulation of keratinocyte adhesion to fibronectin (Figure 2A). One possible explanation for the differences in adhesion to fibronectin in ROCK1-depleted keratinocytes might be a consequence of loss of actinomyosin contractility affecting adhesion complexes. However, under the conditions used in the adhesion assays (1 hour adhesion) we observed no differences in adhesion complex size (data not shown). The role of ROCK1 function in adhesion to fibronectin has been analysed in rat embryo fibroblasts which, when seeded on fibronectin, displayed significantly higher ROCK1 activity than ROCK2 [11]. This led the authors to conclude that the adhesion process has a particular requirement for ROCK1 [11]. This would appear to be consistent with our data in which we see a decrease in adhesion to fibronectin in ROCK1-depleted keratinocytes but it is worth noting that adhesion to fibronectin-coated beads was unaffected in ROCK1-depleted fibroblasts [17]. Alternatively, it might be a consequence of altered fibronectin matrix assembly, as has been observed in fibroblasts [17]. However, this is unlikely to have been a factor in the relatively short period of adhesion used in our assays. It is unclear why adhesion to fibronectin is affected by depletion of ROCK1 or ROCK2 but adhesion to laminin-332 or collagen IV, both of which are known keratinocyte ECM ligands, is unaffected (Figure 2B,C). One possibility is that ROCK1 and ROCK2 regulate expression and/or function of keratinocyte fibronectin receptors (e.g. α5β1 integrin). We have analysed expression and function of the most abundant fibronectin-binding integrin, α5β1, but did not observe any consistent or significant changes in expression or activity (data not shown). This does not rule out the possibility that other fibronectin-binding integrins (e.g. αvβ5) are involved and this is currently being investigated.

Previous data from our laboratory also demonstrated that expression of the constitutively active kinase domain of ROCK2 in human keratinocytes promoted terminal differentiation [4]. Consistent with this, inhibition of ROCK using Y-27632 inhibited keratinocyte differentiation [4]. However, as these experiments involved over-expression of the kinase domain alone, it is possible that the constitutively active mutant would be targeted inappropriately within the cell. Furthermore, Y-27632 inhibits both isoforms of ROCK as well as other kinases such as PRK2 and PKN [13], [24]. The approach we have adopted here, using ROCK isoform-specific RNAi, allows a more detailed analysis of the role of ROCK in keratinocyte terminal differentiation. Interestingly, we found that the two ROCK isoforms have opposing roles in regulating keratinocyte terminal differentiation. Knockdown of ROCK1 promotes keratinocyte terminal differentiation whereas knockdown of ROCK2 has the opposite effect and inhibits keratinocyte terminal differentiation (Figure 3A,C). One possible explanation for these observations is that a ROCK1-interacting protein is involved in promoting differentiation and one candidate here might be RhoE/Rnd3, which binds specifically to ROCK1, and has recently been linked to the stratification and differentiation of human keratinocytes [6], [25].

In summary, we have used RNAi to specifically knockdown ROCK1 or ROCK2 expression in cultured keratinocytes. The data presented in this paper support our previous report of a pivotal role for ROCK signalling in keratinocyte function and extend those observations by reporting that ROCK1 and ROCK2 play distinct and opposing roles in regulation of keratinocyte adhesion and differentiation.

## Materials and Methods

### Reagents and Antibodies

Primary antibodies used were: involucrin (SY-5, Abcam, Cambridge, UK); ROCK1 (H-85, Santa Cruz, USA); ROCK2 (BD Transduction Laboratories, Oxford, UK); tubulin (T6199, Sigma, MO, USA); phospho-MYPT1 (Thr-696-MYPT1, Upstate Cell Signalling Solutions, USA); phospho-MLC (Thr18/Ser19-Myosin Light Chain 2, Cell Signaling Technology, Inc., MA, USA); total MLC (Myosin Light Chain, MY-21. Sigma, MO, USA); keratin 10 (Thermo Fisher Scientific, CA, USA); keratin 5 (Abcam, Cambridge, UK); pan-keratin (Zymed, South San Francisco, CA, USA). Secondary antibodies were purchased from Jackson Immunoresearch (West Grove, PA, USA). All other reagents were purchased from Sigma.

### Cell Culture

SCC12f keratinocytes [22] were co-cultured with mitotically inactivated feeder fibroblasts using the method of Rheinwald and Green as described elsewhere [26]. HaCaT keratinocytes [27] were cultured in DMEM media containing 5% FBS and 1% penicillin/streptomycin. Cell culture medium and reagents were purchased from Invitrogen.

### Plasmids and siRNA Oligos

HaCaT immortalized human keratinocytes were stably transfected with GFP-IRES-shRNAmir constructs purchased from Open Biosystems, UK, to knockdown ROCK1 (RHS4186-97556976 pGinZeo), ROCK2 (RHS4430-98854581 pGipZ) or a nonsense mRNA sequence (Non-Silencing Control) (RHS4346 pGipZ) using the Amaxa HaCaT nucleofection solution V (VCA-1003, Amaxa Inc., MD, USA) according to the manufacturers instructions. Plasmid-expressing HaCaT cells were cultured in 1.5 µg/ml puromycin or 400 µg/ml G418 as appropriate. Cells were lysed at sub-confluence or 2 days post-confluence, as described in the text. SCC12f keratinocytes were transiently transfected with siRNA oligos targeting ROCK1 (ON-TARGETplus siRNA J-003536-06-0020, J-003536-07-0020, Thermo Scientific, USA), ROCK2 (S102223746, S102223753, QIAGEN, UK) or a non-silencing control sequence (S103650325, QIAGEN, UK) using Lipofectamine RNAi MAX reagent (Invitrogen) according to manufacturers instructions.

### Involucrin Immunostaining

SCC12f keratinocytes were fixed with 4% paraformaldehyde in PBS, permeablised with ice-cold methanol and stained with antibodies against involucrin and pan-keratin as described elsewhere [4], [28]. Cells were stained with pan-keratin antibody to ensure only keratinocytes (and not residual fibroblasts) were analysed. Cells were visualized using a Leica DMRB microscope equipped with a Hamamatsu ORCA camera, and images were captured and processed using OpenLab software (Improvision). For each immunostaining, the same exposure time was used to capture images. The percentage of involucrin positive cells compared to the total number of pan-keratin positive cells was calculated and expressed as fold change, with the mean and standard error of 3 separate experiments given. For each experiment a minimum of 3 fields of view (minimum of 150 cells per field) per condition were scored and all experiments were scored blind.

### SDS-PAGE and Western Blotting

Protein lysates were prepared in 3x Laemmli buffer, separated by SDS-PAGE, and immunoblotted as described elsewhere [12]. All experiments were performed on three separate occasions, with representative blots shown.

### Adhesion Assays

Keratinocytes were re-suspended in serum-free medium and plated in 96-well plates (Immulon II; Thermo Electron) previously coated with either full length fibronectin (Sigma) or collagen IV (Sigma) diluted in PBS or a laminin-332-enriched substrate and blocked in bovine serum albumin/PBS for 1 h. After incubation for 1 h, non-adherent cells were removed by washing in PBS, and numbers of adherent cells were assessed by analysis of endogenous hexosaminidase activity [29]. The laminin-332 enriched substrates were prepared as described elsewhere [30]. Statistical analysis was performed using unpaired two-way Student's T-tests.

## Supporting Information

Figure S1 ROCK isoform-specific knockdown regulates cell adhesion to the FIII9-10 integrin-binding domain of fibronectin. Adhesion of HaCaT-NSC, HaCaT-ROCK1-KD or HaCaT-ROCK2-KD to recombinant FIII9-10 was analysed. The mean and standard error of 3 separate experiments are shown. Statistical analysis was carried out using unpaired two-way Student's T-test (** p<0.01, * p<0.05).
(2.65 MB TIF)

Figure S2 ROCK isoform-specific knockdown regulates keratinocyte differentiation. SCC12f keratinocytes were transiently transfected with siRNA oligos to specifically knockdown ROCK1 or ROCK2 As a control SCC12f cells were transfected with a non-silencing control oligo (NSC). Cells were fixed and immunostained to assess involucrin expression. Cells were also stained with a pan-keratin antibody to exclude fibroblasts from the analysis.
(3.67 MB TIF)

## References

[1] Watt FM (2002). Role of integrins in regulating epidermal adhesion, growth and differentiation.. EMBO J.

[2] Adams JC, Watt FM (1989). Fibronectin inhibits the terminal differentiation of human keratinocytes.. Nature.

[3] Jones PH, Watt FM (1993). Separation of human epidermal stem cells from transit amplifying cells on the basis of differences in integrin function and expression.. Cell.

[4] McMullan R, Lax S, Robertson VH, Radford DJ, Broad S (2003). Keratinocyte differentiation is regulated by the Rho and ROCK signaling pathway.. Curr Biol.

[5] Benitah SA, Frye M, Glogauer M, Watt FM (2005). Stem cell depletion through epidermal deletion of Rac1.. Science.

[6] Liebig T, Erasmus J, Kalaji R, Davies D, Loirand G (2009). RhoE Is required for keratinocyte differentiation and stratification.. Mol Biol Cell.

[7] Heasman SJ, Ridley AJ (2008). Mammalian Rho GTPases: new insights into their functions from in vivo studies.. Nat Rev Mol Cell Biol.

[8] Nakagawa O, Fujisawa K, Ishizaki T, Saito Y, Nakao K (1996). ROCK-I and ROCK-II, two isoforms of Rho-associated coiled-coil forming protein serine/threonine kinase in mice.. FEBS Lett.

[9] Leung T, Chen XQ, Manser E, Lim L (1996). The p160 RhoA-binding kinase ROK alpha is a member of a kinase family and is involved in the reorganization of the cytoskeleton.. Mol Cell Biol.

[10] Jacobs M, Hayakawa K, Swenson L, Bellon S, Fleming M (2006). The structure of dimeric ROCK I reveals the mechanism for ligand selectivity.. J Biol Chem.

[11] Yoneda A, Multhaupt HA, Couchman JR (2005). The Rho kinases I and II regulate different aspects of myosin II activity.. J Cell Biol.

[12] Croft DR, Olson MF (2006). Conditional regulation of a ROCK-estrogen receptor fusion protein.. Methods Enzymol.

[13] Ishizaki T, Uehata M, Tamechika I, Keel J, Nonomura K (2000). Pharmacological properties of Y-27632, a specific inhibitor of rho-associated kinases.. Mol Pharmacol.

[14] Shimizu Y, Thumkeo D, Keel J, Ishizaki T, Oshima H (2005). ROCK-I regulates closure of the eyelids and ventral body wall by inducing assembly of actomyosin bundles.. J Cell Biol.

[15] Thumkeo D, Keel J, Ishizaki T, Hirose M, Nonomura K (2003). Targeted disruption of the mouse rho-associated kinase 2 gene results in intrauterine growth retardation and fetal death.. Mol Cell Biol.

[16] Thumkeo D, Shimizu Y, Sakamoto S, Yamada S, Narumiya S (2005). ROCK-I and ROCK-II cooperatively regulate closure of eyelid and ventral body wall in mouse embryo.. Genes Cells.

[17] Yoneda A, Ushakov D, Multhaupt HA, Couchman JR (2007). Fibronectin matrix assembly requires distinct contributions from Rho kinases I and -II.. Mol Biol Cell.

[18] Ito M, Nakano T, Erdodi F, Hartshorne DJ (2004). Myosin phosphatase: structure, regulation and function.. Mol Cell Biochem.

[19] Kawano Y, Fukata Y, Oshiro N, Amano M, Nakamura T (1999). Phosphorylation of myosin-binding subunit (MBS) of myosin phosphatase by Rho-kinase in vivo.. J Cell Biol.

[20] Kimura K, Ito M, Amano M, Chihara K, Fukata Y (1996). Regulation of myosin phosphatase by Rho and Rho-associated kinase (Rho-kinase).. Science.

[21] Levy L, Broad S, Diekmann D, Evans RD, Watt FM (2000). β1 integrins regulate keratinocyte adhesion and differentiation by distinct mechanisms.. Mol Biol Cell.

[22] Rheinwald JG, Beckett MA (1981). Tumorigenic keratinocyte lines requiring anchorage and fibroblast support cultures from human squamous cell carcinomas.. Cancer Res.

[23] Sebbagh M, Renvoize C, Hamelin J, Riche N, Bertoglio J (2001). Caspase-3-mediated cleavage of ROCK I induces MLC phosphorylation and apoptotic membrane blebbing.. Nat Cell Biol.

[24] Davies SP, Reddy H, Caivano M, Cohen P (2000). Specificity and mechanism of action of some commonly used protein kinase inhibitors.. Biochem J.

[25] Riento K, Guasch RM, Garg R, Jin B, Ridley AJ (2003). RhoE binds to ROCK I and inhibits downstream signaling.. Mol Cell Biol.

[26] Rheinwald JG, Green H (1975). Serial cultivation of strains of human epidermal keratinocytes: the formation of keratinizing colonies from single cells.. Cell.

[27] Boukamp P, Petrussevska RT, Breitkreutz D, Hornung J, Markham A (1998). Normal keratinization in a spontanaously immortalized aneuploid human keratinocyte cell line.. J. Cell Biol.

[28] Hotchin NA, Watt FM (1992). Transcriptional and post-translational regulation of β1 integrin expression during keratinocyte terminal differentiation.. J Biol Chem.

[29] Landegren U (1984). Measurement of cell numbers by means of the endogenous enzyme hexosaminidase. Applications to detection of lymphokines and cell surface antigens.. J Immunol Methods.

[30] Carter WG, Ryan MC, Gahr PJ (1991). Epiligrin, a new cell adhesion ligand for integrin α3β1 in epithelial basement membranes.. Cell.

