# Supplementary figures and images for: Distinct Roles for ROCK1 and ROCK2 in the Regulation of Keratinocyte Differentiation
Source: PLoS One. 2009 Dec 4;4(12):e8190. doi: 10.1371/journal.pone.0008190 (PMC2780731; doi:10.1371/journal.pone.0008190)

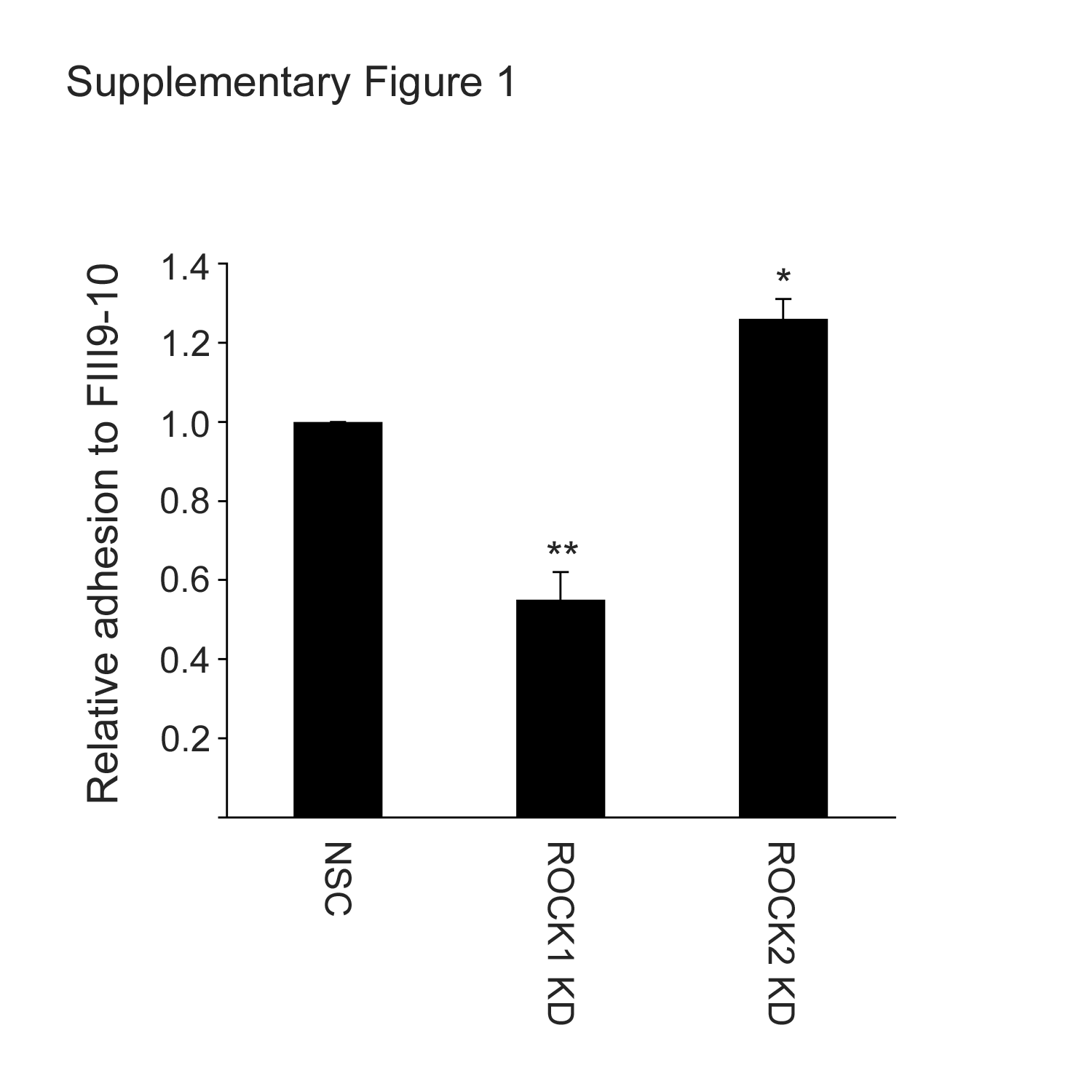

Supplement: Figure S1 — ROCK isoform-specific knockdown regulates cell adhesion to the FIII9-10 integrin-binding domain of fibronectin. Adhesion of HaCaT-NSC, HaCaT-ROCK1-KD or HaCaT-ROCK2-KD to recombinant FIII9-10 was analysed. The mean and standard error of 3 separate experiments are shown. Statistical analysis was carried out using unpaired two-way Student's T-test (** p<0.01, * p<0.05). (2.65 MB TIF) [file pone.0008190.s001.tif]

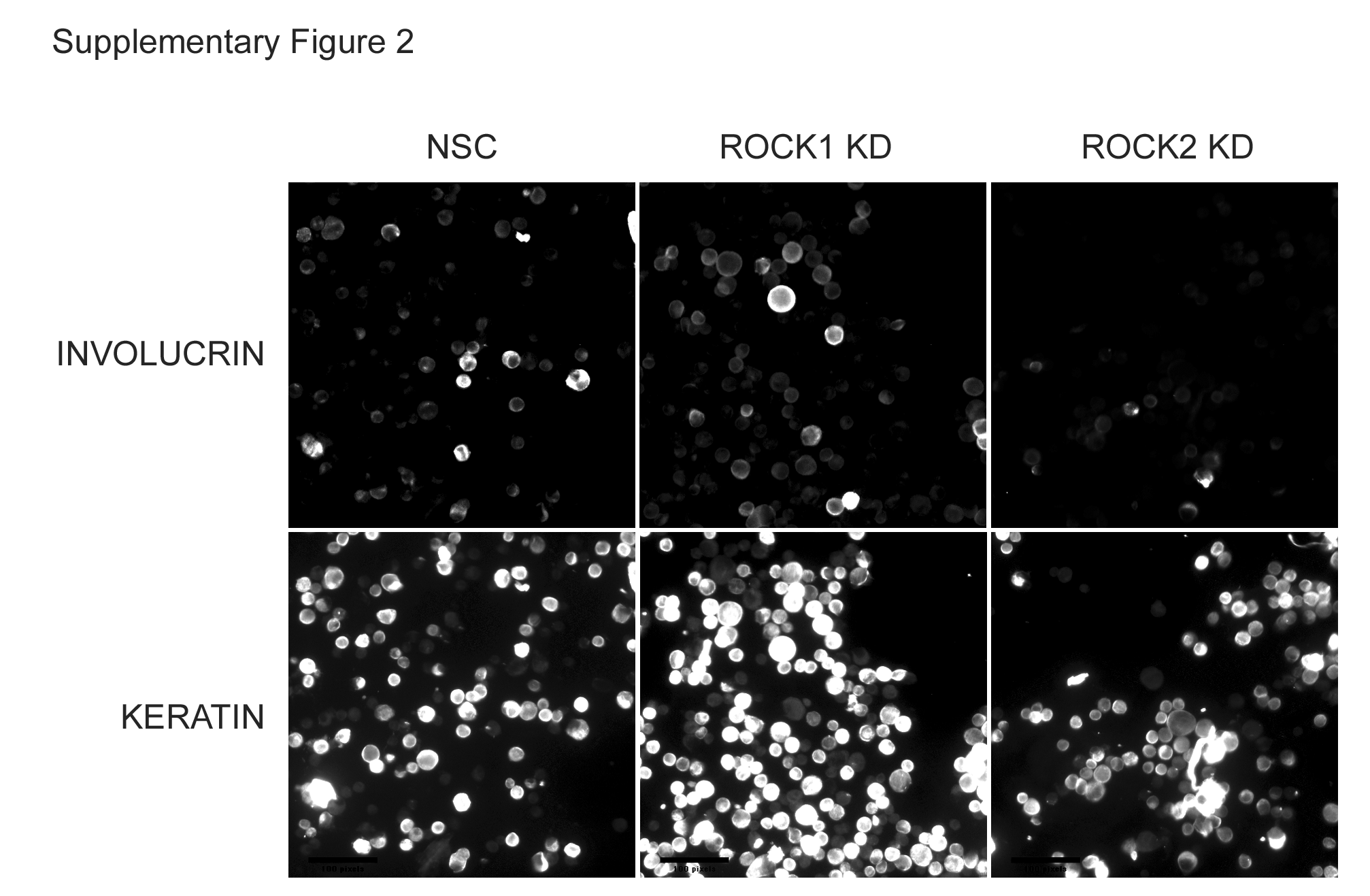

Supplement: Figure S2 — ROCK isoform-specific knockdown regulates keratinocyte differentiation. SCC12f keratinocytes were transiently transfected with siRNA oligos to specifically knockdown ROCK1 or ROCK2 As a control SCC12f cells were transfected with a non-silencing control oligo (NSC). Cells were fixed and immunostained to assess involucrin expression. Cells were also stained with a pan-keratin antibody to exclude fibroblasts from the analysis. (3.67 MB TIF) [file pone.0008190.s002.tif]
